# Comparison of Otsu and an adapted Chan–Vese method to determine thyroid active volume using Monte Carlo generated SPECT images

**DOI:** 10.1186/s40658-023-00609-9

**Published:** 2024-01-08

**Authors:** Jonas Högberg, Christoffer Andersén, Tobias Rydén, Jakob H. Lagerlöf

**Affiliations:** 1 Department of Medical Radiation Physics, and Department of Health, Medicine and Caring Sciences, Linköping University, Linköping, Sweden; 2 Department of Medical Physics, Faculty of Medicine and Health, Örebro University, Örebro, Sweden; 3 Department of Medical Physics and Biomedical Engineering, Sahlgrenska University Hospital, Gothenburg, Sweden; 4 Department of image and Functional Diagnostics, Karlstad Central Hospital, Karlstad, Sweden; 5 Centre for clinical research and education, Region Värmland, Karlstad, Sweden

**Keywords:** Image segmentation, Monte Carlo, SPECT, Thyroid volume, Radioiodine therapy, Otsu, Chan–Vese

## Abstract

**Background:**

The Otsu method and the Chan–Vese model are two methods proven to perform well in determining volumes of different organs and specific tissue fractions. This study aimed to compare the performance of the two methods regarding segmentation of active thyroid gland volumes, reflecting different clinical settings by varying the parameters: gland size, gland activity concentration, background activity concentration and gland activity concentration heterogeneity.

**Methods:**

A computed tomography was performed on three playdough thyroid phantoms with volumes 20, 35 and 50 ml. The image data were separated into playdough and water based on Hounsfield values. Sixty single photon emission computed tomography (SPECT) projections were simulated by Monte Carlo method with isotope Technetium-99 m ($^{\text {99m}}$Tc). Linear combinations of SPECT images were made, generating 12 different combinations of volume and background: each with both homogeneous thyroid activity concentration and three hotspots of different relative activity concentrations (48 SPECT images in total). The relative background levels chosen were 5 %, 10 %, 15 % and 20 % of the phantom activity concentration and the hotspot activities were 100 % (homogeneous case) 150 %, 200 % and 250 %. Poisson noise, (coefficient of variation of 0.8 at a 20 % background level, scattering excluded), was added before reconstruction was done with the Monte Carlo-based SPECT reconstruction algorithm Sahlgrenska Academy reconstruction code (SARec). Two different segmentation algorithms were applied: Otsu’s threshold selection method and an adaptation of the Chan–Vese model for active contours without edges; the results were evaluated concerning relative volume, mean absolute error and standard deviation per thyroid volume, as well as dice similarity coefficient.

**Results:**

Both methods segment the images well and deviate similarly from the true volumes. They seem to slightly overestimate small volumes and underestimate large ones. Different background levels affect the two methods similarly as well. However, the Chan–Vese model deviates less and paired *t*-testing showed significant difference between distributions of dice similarity coefficients (*p*-value $<0.01$).

**Conclusions:**

The investigations indicate that the Chan–Vese model performs better and is slightly more robust, while being more challenging to implement and use clinically. There is a trade-off between performance and user-friendliness.

## Background

The thyroid gland, which is shaped like a butterfly, is located anterior to the trachea and plays a critical role in regulating the body’s metabolism through the production of the hormones Triiodothyronine (T3) and Thyroxine (T4). The volume of the thyroid gland can vary due to natural causes, but it is also influenced by various factors such as gender, age, height, weight, iodine intake, smoking, and environmental circumstances. The relationship between the thyroid gland volume and these characteristics is known to be nonlinear. In a healthy adult without iodine deficiency, the mean sonographic volume of the thyroid gland is estimated to be 7 to 10 ml [1].

The production of T3 and T4 hormones in the thyroid gland depends on the oxidation and binding of iodine to tyrosine. T4, the hormone produced exclusively in the thyroid gland, can be considered a prohormone to T3, as the latter, being the more potent and metabolically active hormone, can be synthesised from T4. Some T3 is secreted directly into the bloodstream, but the largest amount of hormone secreted from the thyroid gland is in the form of T4. The synthesis of T4 to T3 is then carried out by the liver, pituitary gland, and other endocrine organs. Enlargement of the thyroid gland, hyperthyroidism (also known as a goitre), can be caused by various factors including autoimmune diseases (such as Graves’ disease), toxic nodular goitre, cancer, hereditary factors, medication, and diets lacking sufficient amounts of iodine [2].

Hyperthyroidism manifests itself physically as a moderate to severe increase in thyroid volume; a large goitre - 80 g (80 ml) and above - is a contraindication for radioiodine therapy [3, 4]. Thyroid volumes of 40–60 ml may also be considered for surgical procedures, depending on other clinically relevant parameters [5, 6]. Radioiodine treatment has been shown to be effective even within this volume range [7]. Patients with small goitres, which are slightly larger than a normal-sized thyroid gland, are most often treated with antithyroid drugs (ATD), but may later be considered for radioiodine therapy, if 12–18 months of ATD treatment proves ineffective [3, 5, 8].

Different methods are available for assessing the volume of the thyroid gland, including palpation (physical examination), 2D and 3D ultrasound (US), computed tomography (CT), magnetic resonance imaging (MRI), positron emission tomography (PET), and planar scintigraphy (PS). A study by Viduetsky et al. [9] shows that MRI has the best precision with an error of less than 4 %. This volume measurement is useful for surgical planning and monitoring the effectiveness of medication changes. For the individualised planning of radioactive treatment of thyrotoxicosis by Iodine-131 ($^{\text {131}}$I) (radioiodine therapy), single-photon emission computed tomography (SPECT) can be utilised as the imaging modality to determine the active volume of the thyroid gland. SPECT imaging is preferably performed with Technetium-99 m ($^{\text {99m}}$Tc) pertechnetate: the uptake levels of $^{\text {99m}}$Tc pertechnetate and $^{\text {131}}$I are highly correlated, whereas $^{\text {99m}}$Tc SPECT imaging offers better quality, due to lower gamma photon energies, resulting in a lower patient absorbed radiation dose than from $^{\text {131}}$I SPECT imaging [10]. Despite the critical importance of accurate volume determination for the effective prescription of radioactive iodine, there is a lack of consensus among hospitals in Sweden on the preferred method for calculating thyroid volume and $^{\text {99m}}$Tc uptake levels from SPECT or PS images [11]. For the planning of radioiodine therapy of the thyroid, both functional volume, as well as pertechnetate uptake need to be quantified, and SPECT is considered the best way of achieving this [12–15] - neither can 3D shape of active volume be easily described by PS, nor can activity inside the thyroid be distinguished from activity in front of or behind the gland. Thus, thyroid pertechnetate uptake is often overestimated by PS imaging [11, 16].

SPECT images are challenging to segment compared to other tomographic images, such as CT or MRI, due to their low resolution and inherent blurry characteristics. On the other hand, SPECT provides valuable physiological information that is not available from CT or MRI.

A common segmentation approach when SPECT is used is intensity thresholding of the images. This is an arbitrary approach as every patient case is unique, while the threshold values are either fixed or operator-dependent. Apart from being accurate, robust, and operator-independent, a segmentation method for clinical implementation should be easy to use [17].

Two methods that have been successful in determining the volume of other organs are the Otsu method [18] and the Chan–Vese (C–V) model [19–21]. As the volume of the thyroid gland plays a crucial role in radioiodine treatment planning, it is important to assess the accuracy of these methods for segmenting the thyroid gland. The aim of this study is to compare the performance of the Otsu method and the C–V model in calculating the active volume of thyroid glands, taking into account variations in size, activity concentration (AC), heterogeneity, and background AC. The study seeks to determine which method is more accurate and robust in calculating the active volume of thyroid glands, and therefore most suitable for clinical implementation: the Otsu method or the Chan–Vese model.

As radioiodine therapy is considered mainly for moderately sized goitres, typically with a total thyroid gland volume of 20–40 ml and, only in rare cases, 60 - 80 ml, this study focuses on the most relevant thyroid volume range, i.e. 20 - 50 ml [3–6].

## Methods

### Image generation

**Fig. 1:**
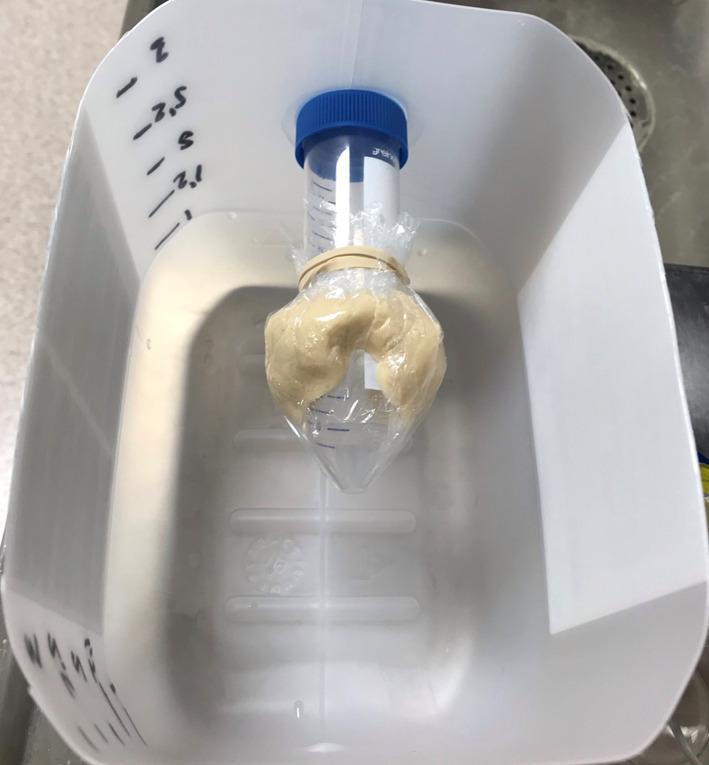
Playdough phantom A playdough fantom fixed on an air-filled plastic tube, mounted inside a water tank. The water level in the tank will reach just above the playdough

To generate images, a computed tomography (CT) was performed of the three playdough thyroid phantoms described in [22], with volumes 20, 35 and 50 ml, using a pixel size of 0.98 by 0.98 mm and slice thickness of 2.5 mm. The formula for the playdough was 38% common salt (NaCl), 36% water, 21% wheat flour and 5% rapeseed oil [22]. The thyroid phantoms were fixed on an air-filled tube (trachea), wrapped in a plastic film, and immersed in water to simulate surrounding tissues (see Fig. 1). The CT images were resampled to 4.42 mm cubic voxels and annotated playdough, water and air, based on thresholding of hounsfield values (600, 0 and -1000, respectively). The threshold values were set to match the true volumes as accurately as possible. The volumes of the annotated playdough phantoms were 20.03, 34.97 and 50.00 ml.

Sixty single photon emission computed tomography (SPECT) projections were generated using the Monte Carlo (MC) method with the $^{\text {99m}}$Tc isotope [23]. The MC algorithm is GPU-based and uses delta scattering, forced interaction, scattering orders (0 to 2) and forced detection for variance reduction. A pre-generated angular response function (ARF), for all angles and energies, was used to model collimator response. For each phantom, primary and scattered radiation were simulated separately for both the background and phantom. The different segments (water, thyroid and air) were assigned attenuation values. The same values as for water were also used for the thyroid voxels. Linear combinations of these SPECT images were created to generate 12 different combinations of volumes and backgrounds, with homogeneous thyroid AC and a hotspot of three different relative ACs, resulting in a total of 48 SPECT images.

**Fig. 2:**
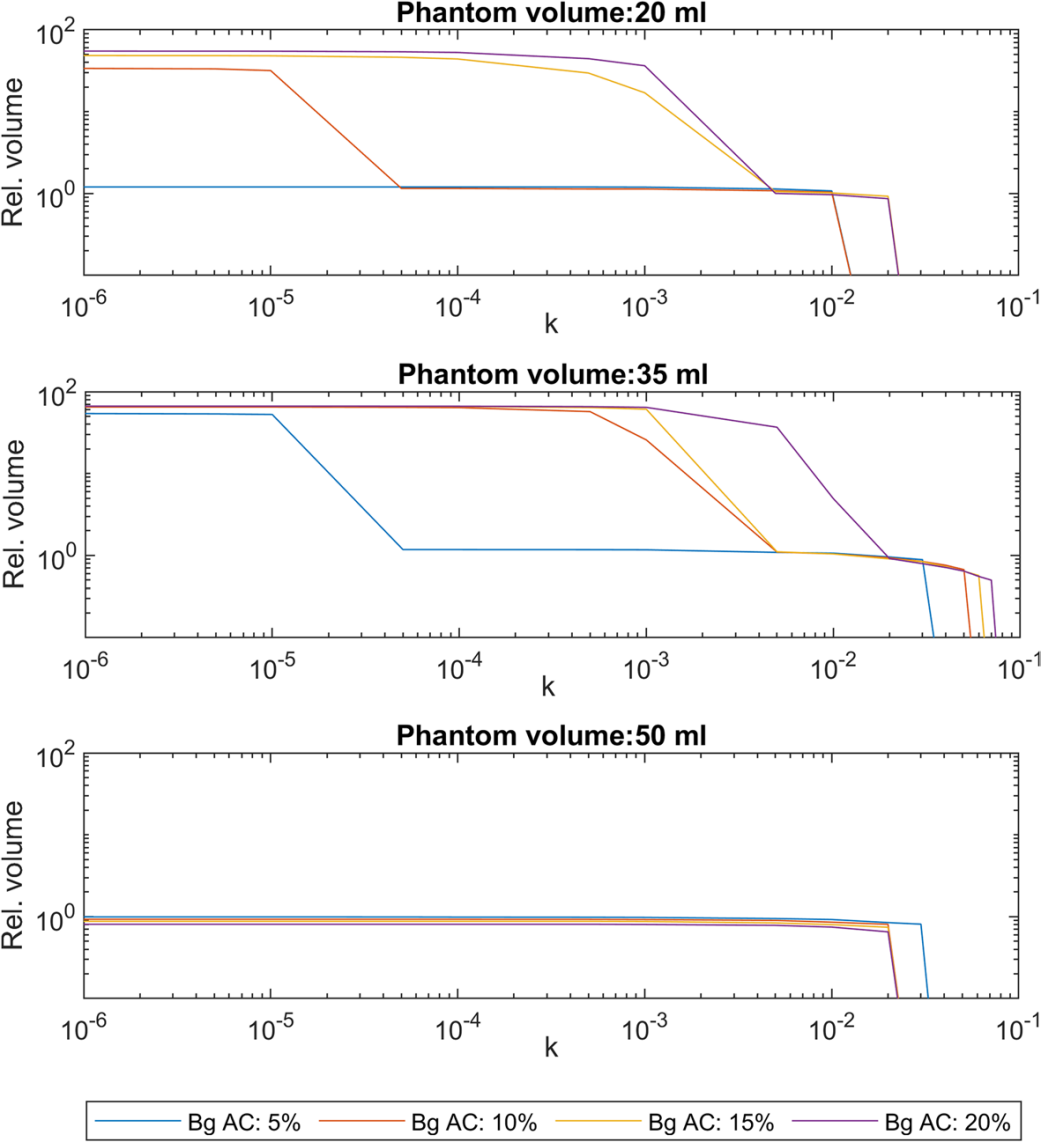
Parameter selection Relative phantom volumes as a function of *k* for phantom volumes 20, 35 and 50 ml and 5 % relative background concentration

**Fig. 3:**
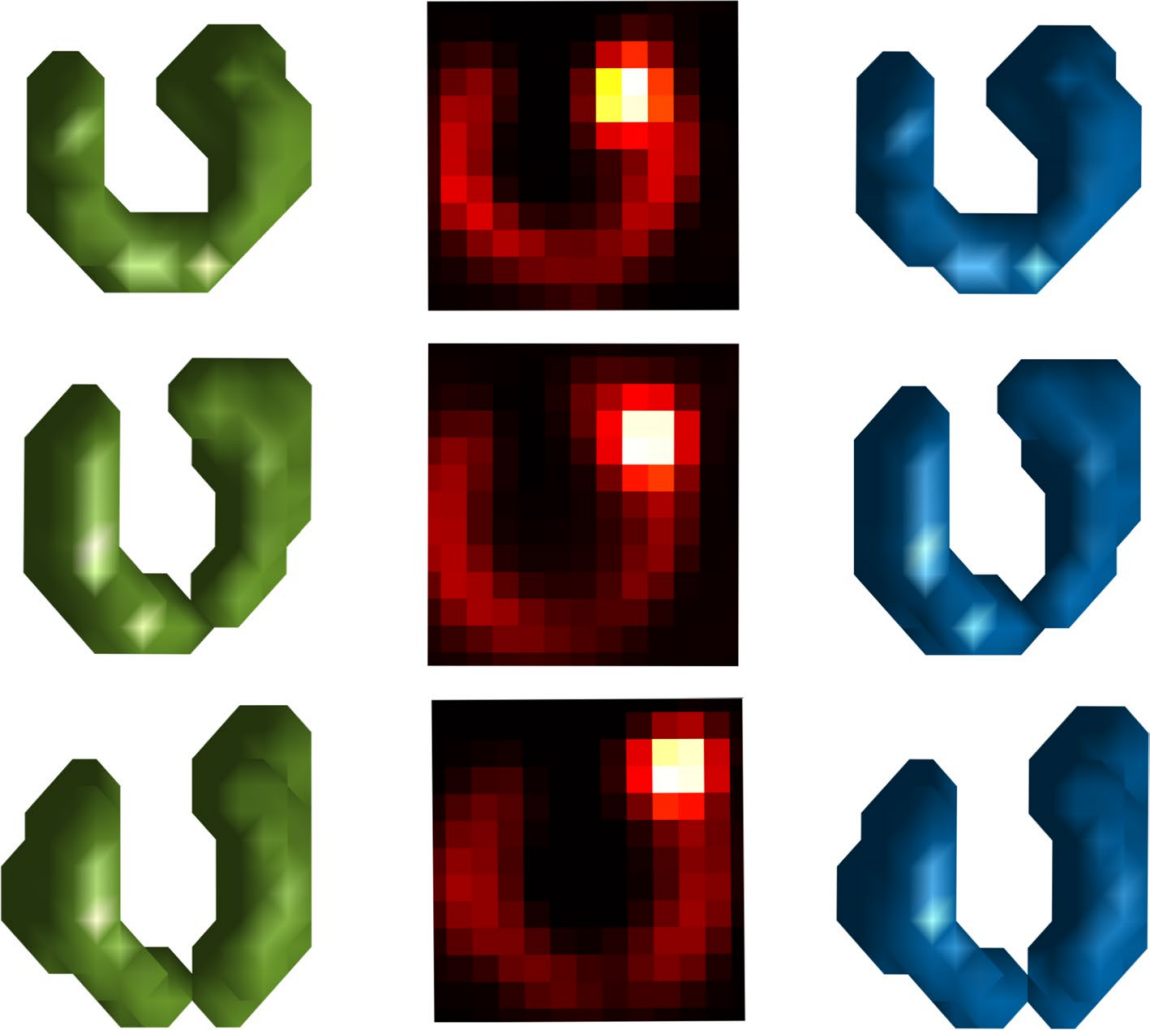
Segmentation example with 200 % hotspot and 10 % background The middle column shows maximum intensity projections of the simulated images for 20 ml (upper), 35 ml(middle) and 50 ml (lower). All images have relative hotspot activity concentration of 200 % and relative background activity concentration of 10 %. The left column shows the corresponding segmentation results from Chan–Vese and the right column shows the results from Otsu

**Fig. 4:**
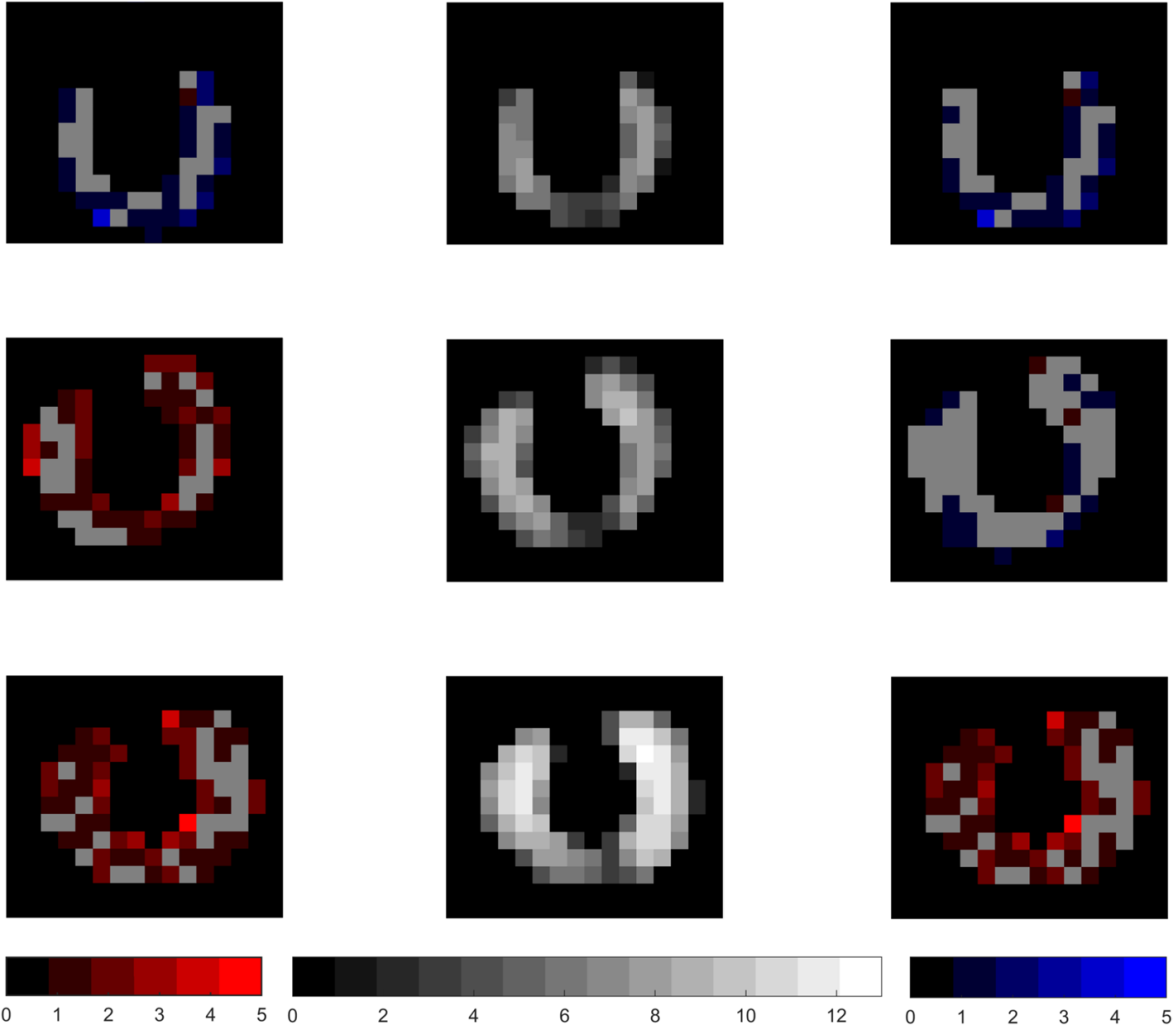
Misclassification example with 200 % hotspot and 10 % background Spatial distribution of misclassified voxels displayed as a sum of voxels in the anterior-posterior direction. The middle column shows the true objects in greyscale, where the greyscale level illustrated the number of superimposed object voxels, for 20 ml (upper), 35 ml(middle) and 50 ml (lower). All images have relative hotspot activity concentration of 200 % and relative background activity concentration of 10 %. The left column shows the sum of segmentation errors from Chan–Vese, with missed object voxels in red and misclassified background voxels in blue, and the right column shows the corresponding results from Otsu. These colour brightnesses describe the number of superimposed erroneous voxels. The misclassifications are displayed on a solid gray background of the true object, for orientation purposes

The relative background levels were set at 5 %, 10 %, 15 %, and 20 % of the phantom AC, while the hotspot activities were set at 100 % (homogeneous case), 150 %, 200 %, and 250 %. Because variance reduction was used in the simulations, Poisson noise, sampled from a Poisson distribution based on the pixel value, was added before reconstruction [24], resulting in a coefficient of variation of 0.8 in the image background of the reconstructed image, in the case of the 20 % background (with the scattering excluded).

A Low energy high-resolution (LEHR) collimator was used, with thickness 35 mm, hole diameter 1,5 mm, septal thickness 0.2 mm, crystal thickness 15.875 mm and intrinsic resolution, full width at half maximum (FWHM), 4.5 mm. The maximum thyroid-detector distance was 100 mm and the system resolution (FWHM) was calculated, according to [25], to be 7.5 mm.

An average of 100,000 counts were recorded in each projection. The matrix size was 128 x 128, with 4.42 mm cubic voxels. The MC-based SPECT reconstruction algorithm Sahlgrenska Academy reconstruction code (SARec) [23] was used for reconstruction, with six subsets and twelve iterations, and no post-filters were applied. SARec is a GPU-based maximum-likelihood expectation-maximisation (MLEM) algorithm that uses the above MC-algorithm to simulate SPECT projections from an estimated activity distribution. For each iteration, the activity estimates are updated through back-projection of the ratio of simulated to measured projection (which in this study is MC-simulated).

### Image pre-processing

For efficient segmentation, it is desirable to have a homogeneous and low background. To achieve this, a background region of interest (ROI) was selected and its mean value was subtracted from the entire image to equate background tissue AC to the surrounding air, which is not radioactive. To prevent potential hotspots from being segmented separately and misclassifying the remaining thyroid tissue as background, a representative ROI of thyroid tissue was outlined, and its mean value was used as an upper limit for values in the image. Although wavelet-based denoising [26] was initially applied, it was found to be irrelevant to the segmentation result and was therefore omitted.

### Image segmentation

The images were imported into Matlab [27] as 8-bit unsigned integer matrices (values between 0 and 255). Two different segmentation algorithms were applied: 1) Otsu’s threshold selection method and 2) the iterative convolution-thresholding method (ICTM) for active contours without edges, developed from the C–V model, by Wang et al. [28]. The results were evaluated based on the relative volume, mean absolute error (MAE), calculated using Eq. 1, standard deviation (SD) (Eq. 2), and dice similarity coefficient (DSC) of the segmented objects (Eq. 3) per thyroid volume. The DSC measures the similarity between the calculated segmentation and the ground truth segmentation, with a score of 1 indicating perfect agreement and a score of 0 indicating no agreement. A paired *t*-test was conducted to see if there was a significant difference ($\alpha =0.05$) between the DSC for the two methods. To investigate the impact of background on segmentation performance, DSC was calculated for background levels between 0 % and 70 % for a 50 ml phantom without hotspot.

1 $$\begin{aligned} \mathrm{{MAE}}= & {} \frac{1}{N}\sum _{i=1}^{N} \vert \hat{V}_{i}-V_{i} \vert \end{aligned}$$

$\hat{V}_{i}$ and $V_{i}$ are the estimated and true volumes, respectively, of each of the N objects.

2 $$\begin{aligned} S=\sqrt{\frac{1}{N-1} \sum _{i=1}^N (\hat{V}_i - \overline{V})^2} \end{aligned}$$

S is the sample standard deviation and $\overline{V}$ is the mean relative volume (MRV).

3 $$\begin{aligned} \mathrm{{DSC}}={\frac{2\vert V \cap \hat{V}\vert }{\vert V\vert +\vert \hat{V}\vert }} \end{aligned}$$

*V* is the volume from the CT images and $\hat{V}$ are the estimates by Otsu or Chan–Vese.

#### Otsu’s method for image thresholding

Otsu’s method is a well-established segmentation algorithm that was developed in the 1970s [18], and is still in use today. This global thresholding algorithm selects the optimum threshold by minimising the weighted within-class variance (or maximising the between-class variance) of the thresholded background and foreground voxels in the image (Eq. 4).

4 $$\begin{aligned} \sigma _{w}^{2}(t)=\omega _{b}(t)\sigma _{b}^{2}(t)+\omega _{f}(t)\sigma _{f}^{2}(t) \end{aligned}$$

$\omega _{b}$ and $\omega _{f}$ are the weights of the background and foreground classes, separated by a threshold t,and $\sigma _{b}^{2}$ and $\sigma _{f}^{2}$ are variances of the classes. The class weight $\omega _{b,f}(t)$ is computed from the 256 bins of the histogram (Eq. 5).

5 $$\begin{aligned} \omega _{b}(t)= & {} \sum _{i=0}^{t-1}p(i) \nonumber \\ \omega _{f}(t)= & {} \sum _{i=t}^{L-1}p(i) \end{aligned}$$

*L* is the total number of greyscale levels and *p*(*i*) are the normalised counts of each bin (greyscale level). The threshold level (*t*) corresponding to the minimum $\sigma _{w}^{2}(t)$ was selected.

#### Chan–Vese model for active contours

The C–V model for active contours without edges is an effective segmentation algorithm suitable for objects without distinct borders, as it does not rely on gradients in image intensity [29]. Additionally, it is tolerant to blurring and noise [29], making it appropriate for medical imaging applications. The algorithm is based on the Mumford and Shah functional for image segmentation, which was first introduced in 1985 [30–32].

This paper will not provide a complete mathematical explanation of the C–V model, but the principle of it is to minimise the weighted sum of the total sums of squares for the object and the background, which corresponds to the total voxel-by-voxel difference from the mean within the object and background, respectively. The weights that are set by the user for the algorithm to converge depend on the heterogeneity of the current image, and the user needs to define an arbitrary initial contour. To encourage a smoother surface, regularisation terms are added to penalise large object volume and surface area. The energy functional to minimise, denoted as $F(c_{1},c_{2},C)$ (Eq. 6),

6 $$\begin{aligned} F(c_{1},c_{2},C)= & {} \mu \cdot area(C) + \nu \cdot volume(inside(C)) \nonumber \\{} & {} +\lambda _{1} \int _{inside(C)} \mid u_{0}(x,y,z)-c_{1} \mid ^{2} \,dx\,dy\,dz \nonumber \\{} & {} +\lambda _{2} \int _{outside(C)}\mid u_{0}(x,y,z)-c_{2}\mid ^{2} \,dx\,dy\,dz, \end{aligned}$$

where *C* is the surface enclosing the object, $\mu$, $\nu$, $\lambda _{1}$ and $\lambda _{2}$ are constants, $u_{0}(x,y,z)$ are individual voxel values, $c_{1}$ and $c_{2}$ are the mean values inside and outside *C*, respectively.

For a three-dimensional problem, it can be solved through various methods. One of these methods, developed by Wang et al., is an efficient, iterative convolution-thresholding method (ICTM) for image segmentation. For this study, a 3D extension of the ICTM was applied to the images. This method utilises a number of approximations to establish a framework for minimising energy functionals. A description and mathematical derivation of this approach is beyond the scope of this paper and can be found in the original papers by Wang et al. [28, 33, 34]. It requires only two parameters to be set by the user, a step length ($\tau$) and $\lambda _{1}$, which, in this setting is a function $\lambda _{1}(k,\tau )$. Hence, in practice, $\lambda _{1}$ is determined by selecting *k* and $\tau$. The value of $\tau$ affects the calculation duration, which is not considered in this study, but not whether the calculations converge, as proved by Wang et al. [34]. A value of $\tau =0.3$, within the range used by Wang et el. was used in all calculations. The value of $\lambda _{1}(k,\tau )$, expressed as $\lambda _{1}(k,\tau )=k \cdot (\pi \cdot \tau ^{-1})^{0.5}$, does determine whether or not the algorithm converges at a reasonable value. In these calculations, the parameter *k* was selected iteratively for each combination of tunour volume and background AC. Figure 2 shows the relative volume as a function of *k* for a few of the phantoms. The selected values are shown in table 1.

**Table 1:** Values of *k* used in the Chan–Vese segmentations, per phantom volume and background AC

Background AC (%)	20 ml	35 ml	50 ml
5	$3\cdot 10^{-4}$	$3\cdot 10^{-4}$	$3\cdot 10^{-4}$
10	$3\cdot 10^{-4}$	$3\cdot 10^{-2}$	$3\cdot 10^{-4}$
15	$1\cdot 10^{-2}$	$6\cdot 10^{-2}$	$3\cdot 10^{-4}$
20	$2\cdot 10^{-2}$	$5\cdot 10^{-2}$	$3\cdot 10^{-4}$

## Results

Figure 3 illustrates segmentation results using the two methods for each of the volumes of the phantoms, using a relative background AC of 10 % and a relative hotspot AC of 200 %. This provides a visual demonstration of how well the segmentation algorithm identifies the thyroid region and separates it from the surrounding background. Both methods describe similar segmentation volumes although slightly different in shape, in this example most prominent for the mid-sized (35 ml) active gland volume. Figure 4 shows the corresponding spatial distribution of misclassified voxels. There does not appear to be any particularly weak areas for either of the methods; the misclassified voxels are distributed across the phantom with no obvious spatial preference.

**Fig. 5:**
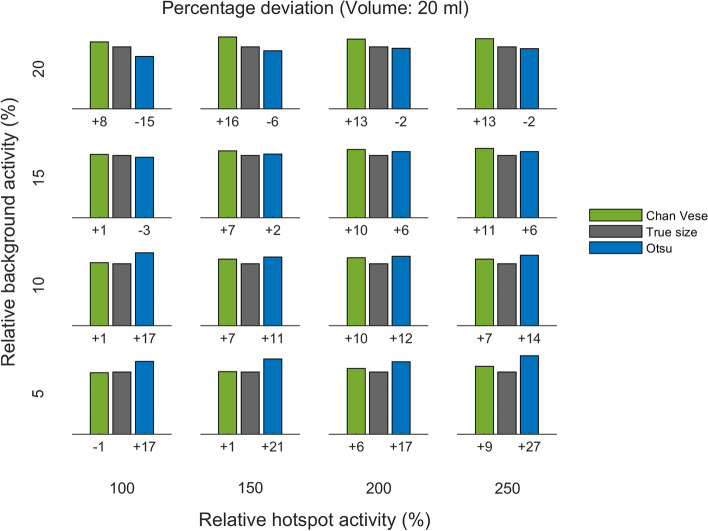
Deviation of size estimates for 20 ml phantom. The calculated volumes for the 20 ml phantom, of Chan–Vese and Otsu method, relative to true size per relative background activity cencentration (rows from bottom to top show 5, 10, 15 and 20 %) and relative hotspot activity concentration (columns from left to right show 100, 150, 200 and 250 %). The figures below each panel show the deviation (in percentages) from true size

**Fig. 6:**
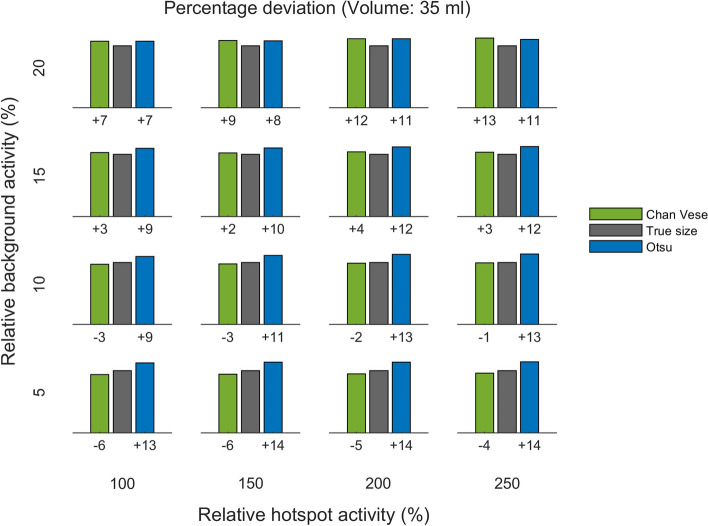
Deviation of size estimation for 35 ml phantom. The calculated volumes for the 35 ml phantom, of Chan–Vese and Otsu method, relative to true size per relative background activity concentration (rows from bottom to top show 5, 10, 15 and 20 %) and relative hotspot activity concentration (columns from left to right show 100, 150, 200 and 250 %). The figures below each panel show the deviation (in percentages) from true size

**Fig. 7:**
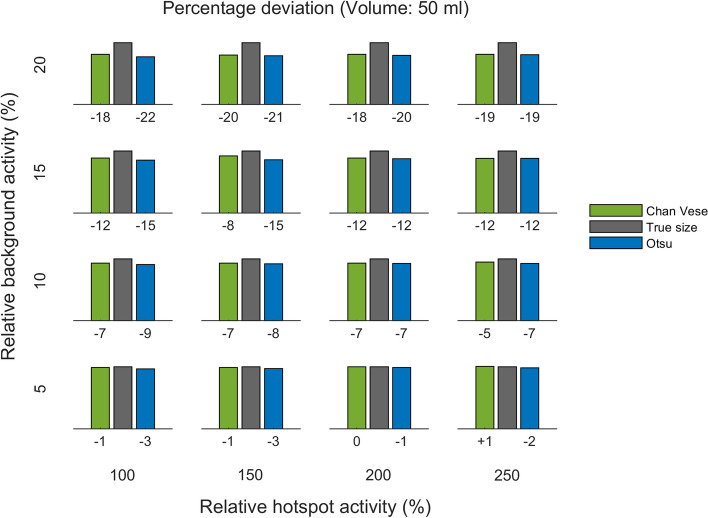
Deviation of size estimates for 50 ml phantom. The calculated volumes for the 50 ml phantom, of Chan–Vese and Otsu method, relative to true size per relative background concentration (rows from bottom to top show 5 %, 10 %, 15 % and 20 %) and relative hotspot activity concentration (columns from left to right show 100 %, 150 %, 200 % and 250 %). The figures below each panel show the deviation (in percentages) from true size

**Fig. 8:**
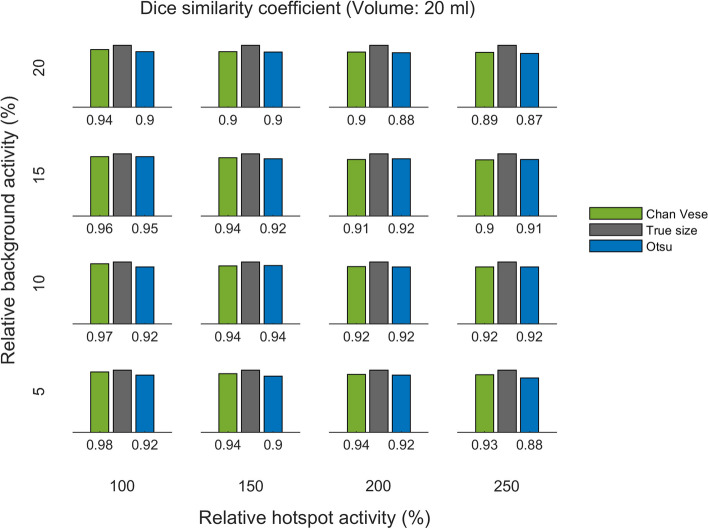
DSC for 20 ml phantom. DSC of Chan–Vese and Otsu method, per relative background activity concentration (rows from bottom to top show 5 %, 10 %, 15 % and 20 %) and relative hotspot activity concentration (columns from left to right show 100 %, 150 %, 200 % and 250 %)

**Fig. 9:**
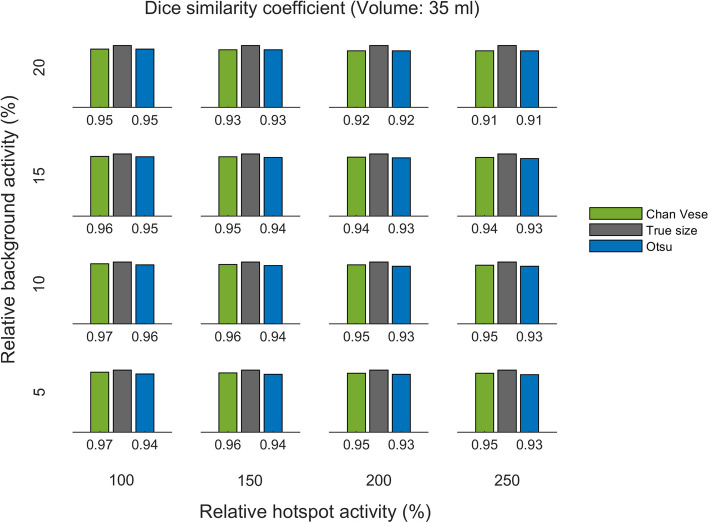
DSC for 35 ml phantom. DSC of Chan–Vese and Otsu method, per relative background activity concentration (rows from bottom to top show 5 %, 10 %, 15 % and 20 %) and relative hotspot activity concentration (columns from left to right show 100 %, 150 %, 200 % and 250 %)

**Fig. 10:**
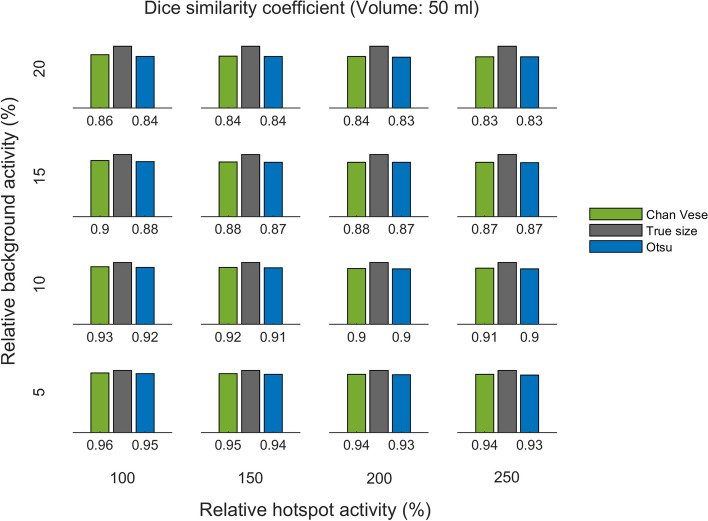
DSC for 50 ml phantom. DSC of Chan–Vese and Otsu method, per relative background activity concentration (rows from bottom to top show 5 %, 10 %, 15 % and 20 %) and relative hotspot activity concentration (columns from left to right show 100 %, 150 %, 200 % and 250 %)

Quantitative results are presented in Figs. 5 , 6, 7, 8, 9 and 10, which show the deviation of size estimates for three different phantoms (20 ml, 35 ml, and 50 ml), calculated using the C–V model and Otsu method. The deviation in estimations of size is presented relative to the true size per relative background AC (rows) and relative hotspot AC (columns). The deviation from the true size is presented as a percentage. Both algorithms seem to have a slight tendency to overestimate small volumes and underestimate large ones and the only visually notable difference between the two models is shown for the mid-size (35 ml) phantom whith low (5 % and 10 %) backgrounds: for this phantom and within this background range the C–V model slightly underestimates, whereas the Otsu method slightly overestimates the active volumes while for small gland with high background the situation is the opposite.

The DSC for the two methods, for the same three phantoms, are shown in Figs. 8, 9 and 10. The DSC is presented per relative background AC (rows) and relative hotspot AC (columns).

Overall, these figures provide a clear visual representation of the performance of the C–V model and Otsu method in terms of size estimates and segmentation accuracy, under different conditions of relative background AC and relative hotspot AC.

**Fig. 11:**
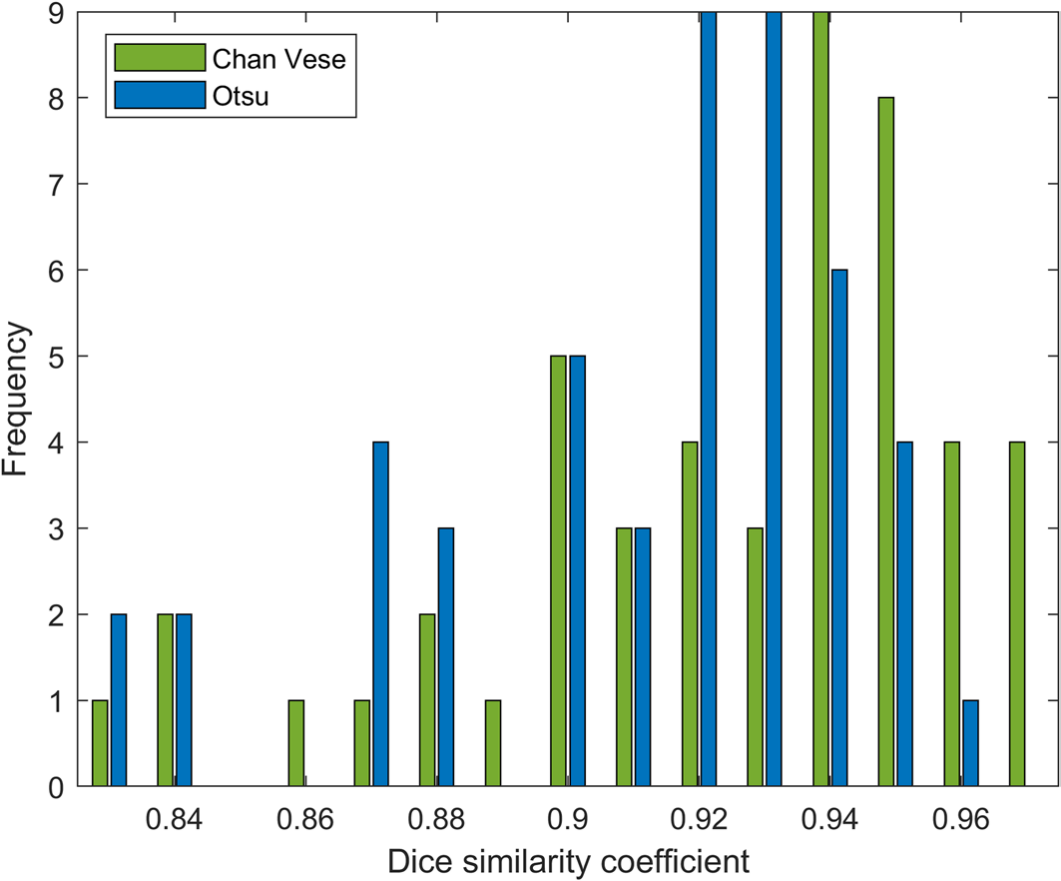
DSC distribution total distribution of DSC of Chan–Vese and Otsu method

**Fig. 12:**
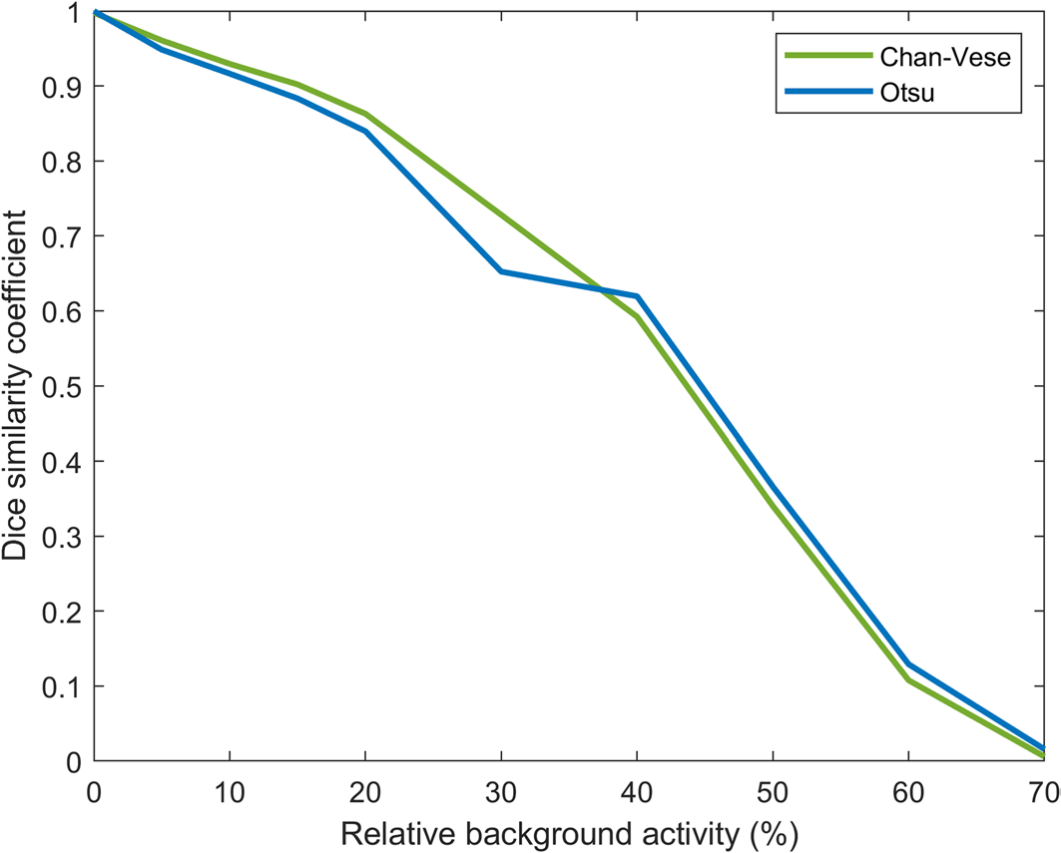
Background impact DSC as a function of background level for a homogeneous 50 ml phantom

The total DSC distributions are shown in Fig. 11. A paired *t*-test showed that the difference between the distributions is significant (*p*-value $<0.01$), with Chan–Vese being the more accurate algorithm. The variation of DSC between different background levels is shown in Fig. 12.

Tables 2 and 3 present the mean absolute errors, mean relative volumes, and standard deviations of the estimations. In total, the C–V model has slightly lower values for both MAE and MRV than the Otsu method.

**Table 2:** Mean absolute errors, mean relative volumes and standard deviations for the Chan–Vese segmentations, per phantom volume

Volume (ml)	MAE (%)	MRV	SD
20	7.6	1.07	0.048
35	4.9	1.01	0.060
50	9.2	0.91	0.070
20-50	7.2	1.00	0.091

**Table 3:** Mean absolute errors, mean relative volumes and standard deviations for the Otsu segmentations, per phantom volume

Volume (ml)	MAE (%)	MRV	SD
20	11.1	1.07	0.073
35	11.3	1.11	0.021
50	11.1	0.89	0.069
20-50	11.2	1.03	0.059

## Discussion

**Table 4:** Otsu threshold values for each case

Volume	Background AC (%)	Hotspot 0	Hotspot +50	Hotspot +100	Hotspot +150
20	5	80	80	87	92
	10	82	97	97	97
	15	91	91	91	91
	20	107	107	107	107
35	5	103	103	103	103
	10	98	110	110	110
	15	97	97	97	97
	20	89	107	89	107
50	5	80	80	80	80
	10	77	71	77	77
	15	73	73	73	73
	20	80	70	70	70

C–V model and Otsu method were selected because they are well-tried [19–21] and highly efficient segmentation methods that are not dependent on gradients (sharp edges) [35], which are prone to segmentation image noise and artefacts when input data contain diffuse volume boundaries [36, 37], as is the case in SPECT imaging. (Table 4)

Both methods (Otsu and C–V) manage to segment the images well, and they also tend to deviate in a somewhat similar way from the true volumes. Although not statistically proven, both algorithms seem to have a slight tendency to overestimate small volumes and underestimate large ones.

Although there are some differences in the results, the overall behaviour of the models does not differ substantially. Nevertheless, the, by Wang et al. [28], adapted C–V model performed slightly better and is the more complex of the two, but it requires *k* to be chosen with care, while Otsu requires no other information than what may be extracted from the image itself. As shown in Fig. 2, the choice of correct parameter value could be a complicated task; this is true in particular for smaller thyroid volumes, as different background activity concentrations are shown to cause different segmentation volume outputs for the same choice of *k*. Albeit low in complexity, the Otsu method has proved to be a robust segmentation tool [21, 38].Given the similar performance, the simplicity and straightforwardness of the Otsu method make it a powerful competitor.

### Limitations

A major limitation of this study was the relatively low number of simulated cases. In addition to this, the lack of heterogeneity of the thyroid phantoms (in addition to the hotspots) and background AC, as well as the lack of variation in thyroid shape and the shape and composition of the background phantom, as this affects the attenuation properties. Although the physical thyroid phantom contains substantial ammounts of the relatively high-Z components Chlorine ($_{17}$Cl) and Sodium ($_{11}$Na), this did not affect the results, as the attenuating properties of the digitised thyroid phantom were set to be water equivalent. Due to the limited resolution of the SPECT images, some voxels will contain image information from playdough (thyroid gland) and water (background). This kind of partial volume effect limits the accuracy of the volume determination. For a thresholding method, such as the Otsu method, the partial volume effect is expected to cause the relative volume overestimation as well as the underestimation of maximum AC being most prominent for the small active volumes [39]. Furthermore, the actual study showed the lowest Otsu segmentation performance, resulting in a volume overestimation as high as 27 %, for the smallest (20 ml) thyroid volume, the highest hotspot AC (250 %) and the lowest background (5 %), i.e. for the highest AC contrast. The explanation for the largest volume overestimation at the highest and not at the lowest contrast is probably the mentioned partial volume effect, prominent for small volumes, combined with the fact that the Otsu method biases towards the image component (either background or foreground) with the largest within-class variance [40, 41]; a lower background in a noisy context, i.e. in SPECT imaging, will have a larger within-class variance than a higher background with the same absolute noise level, as the spectrum will have a lower weight at the lower end for the higher background. This means that the bias will be the strongest and thus the threshold be lowered the most for the lowest background, resulting in the largest relative volume overestimation for this particular case, in line with the result of the actual study. In conclusion, smaller volumes are harder to segment and the thresholding nature of the Otsu method makes the task of segmenting small volumes more challenging for Otsu compared to the C–V model, the latter not relying on thresholding and thus expected to be less sensitive to partial volume effects.

Since simulated patients do not move at all, these images are free from artefacts, caused by movements or physiological dynamics, which always occur to some extent in vivo. These effects are difficult to quantify but should be of little importance.

### Future work

A more thorough investigation of noise level and phantom heterogeneity might be desirable but was considered beyond the scope of this study, as it would be necessary to take methods for homogenisation and noise reduction into consideration to provide optimal conditions for the algorithms. This would be interesting future work, as would a study on the impact of irregularity of phantom shapes, as well as various reconstruction and post-filtration settings. With sufficient access to clinical data, a comparison with AI-based segmentation would also be interesting. With the final objective being the delivery of a correct radiation dose, a dosimetric evaluation of the different methods would be a highly relevant continuation of this work.

## Conclusions

There is a lack and a need for consistency in the determination of thyroid active volume in nuclear medicine images. Even though the, by Wang et al. [28], adapted C–V model drew the longest straw when it comes to accuracy, this study shows a good performance of both methods investigated, under the given circumstances. In terms of autonomy, simplicity and robustness, Otsu is the preferred method. Ultimately it becomes a trade-off between performance and user-friendliness.

## Data Availability

The codes used during the current study and the datasets generated during and/or analysed during the current study are available from the corresponding author on reasonable request.

## Abbreviations

AC: Activity concentration
ARF: Angular response function
ATD: Antithyroid drugs
CT: Computed tomography
C–V: Chan–Vese
DSC: Dice similarity coefficient
ICTM: Iterative convolution-thresholding method
$$^{{131}}$$I: Iodine-131
MAE: Mean absolute error
MLEM: Maximum-likelihood expectation-maximisation
MRV: Mean relative volume
MC: Monte Carlo
MRI: Magnetic resonance imaging
PS: Planar scintigraphy
PET: Positron emission tomography
ROI: Region of interest
SARec: Sahlgrenska academy reconstruction code
SD: Standard deviation
SPECT: Single photon emission tomography
$$^{{99m}}$$Tc: Technetium-99 m
T4: Thyroxine
3D: Three-dimensional
T3: Triiodothyronin
2D: Two-dimensional
US: Ultrasound
